# iCTNet: A Cytoscape plugin to produce and analyze integrative complex traits networks

**DOI:** 10.1186/1471-2105-12-380

**Published:** 2011-09-26

**Authors:** Lili Wang, Pouya Khankhanian, Sergio E Baranzini, Parvin Mousavi

**Affiliations:** 1 School of Computing, Queen's University. 25 Union Street, Goodwin Hall, Queen's University. Kingston, Ontario K7L 3N6, Canada; 2 Department of Neurology, University of California San Francisco. 513 Parnassus Ave. Room S-256. San Francisco, CA, 94143. USA

## Abstract

**Background:**

The speed at which biological datasets are being accumulated stands in contrast to our ability to integrate them meaningfully. Large-scale biological databases containing datasets of genes, proteins, cells, organs, and diseases are being created but they are not connected. Integration of these vast but heterogeneous sources of information will allow the systematic and comprehensive analysis of molecular and clinical datasets, spanning hundreds of dimensions and thousands of individuals. This integration is essential to capitalize on the value of current and future molecular- and cellular-level data on humans to gain novel insights about health and disease.

**Results:**

We describe a new open-source Cytoscape plugin named iCTNet (integrated Complex Traits Networks). iCTNet integrates several data sources to allow automated and systematic creation of networks with up to five layers of omics information: phenotype-SNP association, protein-protein interaction, disease-tissue, tissue-gene, and drug-gene relationships. It facilitates the generation of general or specific network views with diverse options for more than 200 diseases. Built-in tools are provided to prioritize candidate genes and create modules of specific phenotypes.

**Conclusions:**

iCTNet provides a user-friendly interface to search, integrate, visualize, and analyze genome-scale biological networks for human complex traits. We argue this tool is a key instrument that facilitates systematic integration of disparate large-scale data through network visualization, ultimately allowing the identification of disease similarities and the design of novel therapeutic approaches.

The online database and Cytoscape plugin are freely available for academic use at: http://www.cs.queensu.ca/ictnet

## Background

In recent years, the availability of high throughput datasets from a variety of biological sources has prompted the creation of a multitude of databases that significantly facilitate biomedical research. In parallel, network biology has emerged as a powerful paradigm to visualize and analyze large data ensembles in novel ways with unparalleled flexibility [1]. More recent applications of this approach have enabled a detailed look at the genetic landscape of complex human phenotypes [2]. In 2007, Goh et al. reported the first human disease network and provided a novel view of the genetic relationship among diseases [3]. Subsequently, more complex approaches that included the integration of quantitative trait loci, gene expression, and clinical phenotypic data were used to construct disease similarity networks [4,5]. Another pioneering study summarized the application of protein networks for network-based classification of diseases [2] and integration of drug targets and disease gene products led to the field of systems pharmacology [6,7]. Overall, the availability of large-scale datasets has prompted efforts to integrate data with the ultimate goal of providing systematic insights into complex traits.

Recently, multiple databases were elegantly combined to explore gene-disease associations [8]. While this is a useful tool to visualize relationships among phenotypes and study disease-related genes, the obtained networks are limited to only this type of interaction. Here we present iCTNet (integrated Complex Traits Networks), a tool to create and analyze human complex traits networks that assembles and integrates information from genome-wide association studies, protein-protein interactions, tissue expression, and drug targets with the goal of identifying novel relationships across several domains that may assist in elucidating a new classification, pathogenic mechanism, or treatment for common human traits. To the best of our knowledge, iCTNet constitutes the first effort to integrate multiple layers of information as multi-partite networks thus enabling systematic analysis of human complex traits.

## Implementation

The software Cytoscape is a popular open source platform for complex network analysis and visualization that allows for a wide variety of applications through the development of task-specific plugins [9]. iCTNet is implemented in Java as a Cytoscape plugin and provides a user-friendly interface to search, view, and analyze genome-scale biological networks for more than 200 common human diseases and traits. Integration of these data sources results in four types of nodes (i.e. protein/gene/SNP, trait, tissue, and drug), and five types of edges connecting these nodes (i.e. phenotype-SNP, protein-protein, phenotype-tissue, tissue-gene, and drug-gene). In iCTNet, the basic level of analysis is the disease-gene relationships. Once those edges are specified (at any valid p-value threshold) any additional relationship can be incorporated. *We use the terms SNP/gene/protein interchangeably because of the correspondence between SNPs and genes -- more specifically, the most significantly associated SNPs define a gene-trait edge and gene products (proteins) are used in the interaction networks*. Data sources and their integration (Table 1) are described in the following subsections. Once installed, iCTNet is available from the plugin directory of Cytoscape. Through this plugin the user can connect to the database and fetch multiple sources of data for subsequent analysis within the plugin itself or the Cytoscape environment. For example, we have implemented two published algorithms (random walk with restarts and PRINCE) that use network topological characteristics in the protein interaction network to prioritize candidate genes. In essence, the core of both algorithms is similar (i.e. a random walker). The main difference is the type of input data and the type of connections each algorithm can analyze. Random walk with restarts is implemented to work with protein-protein interaction networks only while PRINCE has been extended to work over the entire network with up to 5 different types of connections.

**Table 1:** Data sources and distribution

	Genes (proteins)	Diseases	Drugs	Tissues
Genes (proteins)	39,137^a^	12,539^b^	6,053^c^	73,131^d^
Diseases	12,539^b^	NA	NA	285^e^
Drugs	6,053^c^	NA	NA	NA
Tissues	73,131^d^	285^e^	NA	NA

^a ^HPRD/UCSD;^b ^GWAS Catalog;^c ^DrugBank;^d ^Unigene;^e ^manual curation.

### Data sources

#### Phenotype-SNP association

Phenotype-SNP associations were collected from published GWAS articles and mapped to the closest gene using build 37 of the human genome reference. Our database also contains results from 118 GWAS published before March 1, 2008 [10], and data from the GWAS catalog [11] (Jan 2011). While data from the GWAS catalog only contains associations at a p-value of 10^-6 ^or better, Johnson et al. compiled all available results as reported by each author (hence, p-values of up to 0.05 are reported). An advantage of using GWAS data is the availability of p-values, a measure of confidence for each reported association. Fisher's meta p-value [12] was calculated when the same phenotype-SNP association was reported in different studies. iCTNet contains more than 6,000 trait-SNP associations at a significance level of 10^-4^, and more than 2,000 at 10^-8 ^(Table 2).

**Table 2:** Number of disease-gene associations at different GWAS cutoff values

Cutoff	Disease-gene associations
No filter	12,539
10^-4^	6,284
10^-5^	4,626
10^-6^	3,163
10^-7^	2,459
10^-8^	2,026
10^-9^	1,696
10^-10^	1,489

#### Protein interactions

More than 33,000 interactions among 12,000 proteins were downloaded from the human protein reference database (HPRD, R.8) [13]. In addition, protein (TF) -DNA interactions were incorporated by extracting transcription factor binding sites (TFBS) information from the UCSC Genome Browser v.18 [14]. Protein-DNA interactions were defined as directed edges linking TF and their target genes. Edges were derived from high confidence TFBS (ZScore > 4.0) located within 5,000 bp of the transcriptional start site of the nearest gene.

#### Tissue information

To refine phenotype-SNP association, we incorporated information on genes expressed in specific tissues. This information was downloaded from HPRD and included 13,337 genes, 579 tissues and 10,486 tissue-gene connections. We curated and pooled the tissues into 38 categories to standardize the naming conventions in various sources, and filtered "unknown" gene symbols, resulting in 7,260 genes and 43,206 tissue-gene edges. We also manually curated the disease-tissue association for the phenotypes present in iCTNet. The final disease-tissue subnetwork has 27 pooled tissues, 247 phenotypes and 281 edges.

#### Drug-gene interactions

Drug-target information was obtained from DrugBank v.2.5 [15]. Gene symbols in DrugBank were matched to UCSC nomenclature [14]. This layer has 2,402 drugs and 1,739 gene nodes, linked by 5,989 edges.

### Candidate gene prioritization

Two prioritization algorithms were implemented in iCTNet whose core computation is based on the flow of information across the protein interaction network: i) random walk with restarts [16] and ii) network propagation (PRINCE) [17]. The core of both implemented algorithms is similar (they are both random walk methods). As explained in the original manuscripts [16,17], these methods are implemented and based on the following equation:

$$p^{t+1}=\left(1-r\right)Wp^{t}+rp^{0}$$

where an iterative walker's transition in the network is explained and where *p^t+1 ^*is the vector holding the scores of the nodes at time step *t *+ 1, *W *is the normalized adjacency matrix of the network, *p^t ^*is a vector holding the score of the nodes at the previous time step *t*, and *r *is the restart rate ranging from 0 to 1. In both methods, the walker begins with starting nodes and extends to randomly selected neighbors in the network. The restart ratio represents the probability of the transition to jump back to starting nodes at every time step. In other words, the transition will reach farther nodes in the network with small restart ratio; otherwise, the walker will be trapped at starting nodes if the restart ratio is 1.

The main difference between PRINCE [17] and the random walk method [16] is the input data *p*^0 ^and the adjacency matrix *W*. In random walk with restarts, the initial vector *p*^0 ^was constructed such that equal probabilities were assigned to the starting nodes. Next, all genes with GWAS p-values are classified as either "associated" or "candidate" based on a user-selected threshold. This algorithm measures the closeness of potentially associated (candidates) to confirmed (associated) genes within the global protein network, and ranks candidate genes for further biological investigation. As for the PRINCE algorithm, the original version takes as input a disease similarity matrix (arbitrarily defined), and a protein interaction network. PRINCE then uses a network propagation-based algorithm to infer a strength-of-association scoring function and exploits the prior information on causal genes for the same disease or similar ones. This scoring is used in combination with a PPI network to infer protein complexes that are involved in a given disease. We modified the algorithm to work with unweighted protein-protein interactions and extended it to include all types of network interactions supported by iCTNet. In addition, instead of using an arbitrarily defined disease similarity matrix, our implementation of PRINCE uses true disease associations as defined by a user-selected p-value threshold. A genetic similarity network is then created from GWAS data. Finally, the association of candidate genes with a given phenotype is prioritized via network propagation as originally described. The complexity of both methods is *O*(*tn*^2^), where n is the number of nodes in the network, and t represents the number of time-steps. The run times depend on the number of truly associated genes, their associated strength (p-value), and the number of connections among their protein products in a network.

## Results and Discussion

**Figure 1:**
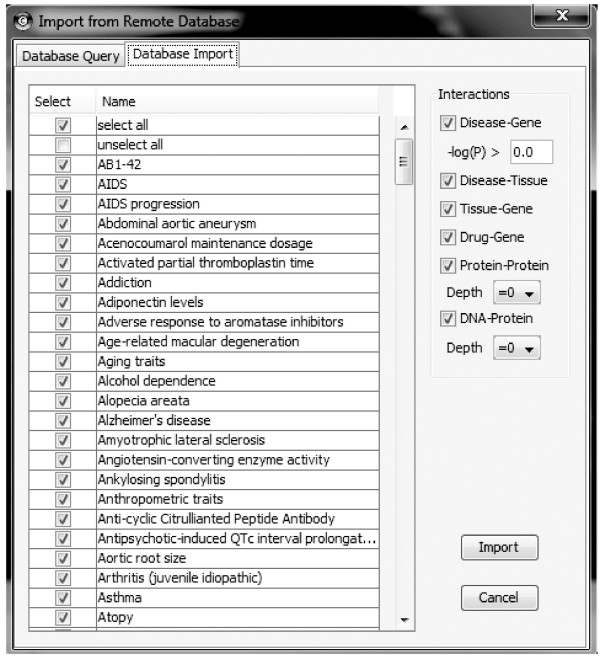
**Screenshot of iCTNet**. Genetic association data for more than 200 traits and diseases are available to download from the iCTNet database at a user-selectable significance threshold (-Log_10_(p)). In addition, the user can choose to download disease-tissue, tissue-gene, and drug-gene interactions by simply ticking a checkbox. Protein-protein and protein-DNA interactions can also be downloaded at different degrees of separation (ds) or distance. Choosing a distance ds = 0 only downloads direct disease-gene associations, and any existing interaction among associated gene products (protein-protein). A distance ds = 1 will also include the first neighbors of genes directly associated.

**Figure 2:**
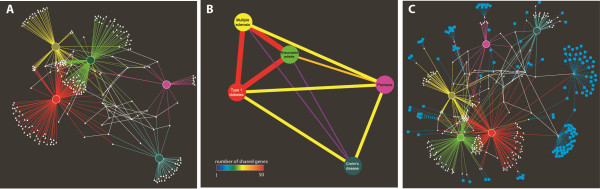
**A network of five common autoimmune diseases**. **A**. Disease-gene interaction network (ds = 0) for five common autoimmune diseases. Each disease has unique and shared associations. RA, T1D, and MS are closely related both through HLA and non-HLA associated genes. **B**. A simplified version of the network shown in **A**, using the "create similarity net" feature of iCTNet. In this representation, diseases are connected by edges of a color proportional to the number of shared genes. **C**. Same network as in **A **with drug-target interactions. Colored circles represent diseases (MS = yellow, T1D = red, RA = green, Ps = magenta, CD = teal), white triangles represent genes, and cyan round squares represent drugs. Disease-gene interactions are colored according to the disease. Protein-protein and DNA-protein interactions are shown as white edges. Drug-gene interactions are represented as cyan dashed edges.

Once installed in the appropriate directory (cytoscape/plugins) iCTNet is available from the Plugins menu within Cytoscape. Through the initial menu, the user can choose to load a network from a local file, or download a network from the iCTNet database. The user can then create a similarity network or perform a series of network analyses. A typical user will choose to start by downloading a network from the iCTNet database. The "Database query" option offers the possibility to query the database for a particular gene, disease, or tissue. Alternatively, the user can choose to directly download any of these datasets to the Cytoscape environment (Figure 1). For example a user could quickly create a gene-disease network with 5 common autoimmune diseases (Type 1 diabetes, T1D; rheumatoid arthritis, RA; multiple sclerosis, MS; Crohn's disease, CD; and psoriasis, Ps) at a p-value of 10^-8 ^or better (for this specific example only disease-gene, protein-protein and protein-DNA interactions were retrieved). This will create a network with 239 nodes (5 nodes of type = disease and 234 nodes of type = gene) and 395 edges. The user has the choice of downloading just the directly associated genes as well as their neighbors at different degrees of separation (ds). A layout of this network (ds = 0) using the built-in edge-weighted spring-embedded algorithm places RA, MS, and T1D next to each other, while Ps and CD are placed further apart (Figure 2A). The proximity among RA, T1D, and MS is consistent with their strong HLA association, while much less evidence of such an association is currently available for Ps and CD. This visualization highlights both the amount of shared genes as well as disease-specific associations. Since all interactions downloaded through iCTNet are from published articles, each disease-gene edge has a PubMed ID as an attribute and the article can be retrieved directly within Cytoscape using the Edge Linkout feature.

A useful feature of iCTNet is the ability to create a similarity network on any existing network using any type of node. This function replaces indirect connections in the bi-partite network (in this case diseases are connected though genes) thus creating a simpler display. For example, a similarity network of the autoimmunity graph shown in Figure 2A is shown in Figure 2B. This feature is particularly useful when handling very large networks. The color of each edge is proportional to the number of genes shared by each disease (using a heatmap coloring scheme). Another key component of iCTNet is the availability of drug-target relationships. Figure 2C shows the same autoimmunity network in which proteins that are drug targets are linked to drugs (blue nodes) as described in DrugBank. A straightforward advantage of this multidimensional display is that it may identify drugs that are effectively used for one disease as a plausible alternative for another disease genetically associated to the same drug target.

iCTNet also provides two candidate prioritization algorithms that take full advantage of the underlying protein interaction network. Both the random walk and PRINCE algorithms take a set of "associated" genes (genes with association p-value below a user-selected threshold) and perform searches through the entire protein interaction network. This is a powerful way to identify a set of candidate genes that even if their association p-value is modest, their position in the protein ensemble makes them suitable candidates for further follow-up. To the best of our knowledge a head-to-head comparison of these algorithms under extensive range of parameters has not been performed. Thus, we are unable to recommend the use of a particular algorithm, and instead encourage the user to test them under different experimental scenarios.

Previous studies that explored the relationship between genes and diseases on a large scale [3,8] were based on manually curated databases such as the Online Mendelian Inheritance in Man (OMIM) [18]. While data from OMIM is readily accessible, the relationships between genes and diseases from its GeneMap do not strictly represent susceptibility loci, but in some cases also refer to progression or pharmacogenomics effects. In contrast, iCTNet incorporates phenotype-SNP associations from the genome-wide association studies (GWAS) Catalog database (http://www.genome.gov/gwastudies). When multiple sources of information implicate a given gene with a trait, p-values from those studies were combined into a meta p-value. As a result, each disease-gene interaction (edge) in iCTNet has a quantitative value approximately equal to the -Log_10 _of the association p-value (-Log_10_(p)). This strategy also enables the iCTNet user to filter results based on a given significance threshold. Another distinctive feature of iCTNet is that multi-partite networks can be created by combining up to four classes of nodes (disease, gene, drug, and tissue) with up to five classes of edges (protein-protein, protein-DNA, disease-gene, drug-gene, and tissue-gene).

In summary, here we present a database and Cytoscape plugin for the integration of different high-throughput datasets. iCTNet represents a new family of applications that are designed to integrate and analyze disparate data sources, a key pillar in the new paradigm of systems biology.

## Conclusions

iCTNet is a powerful plugin for Cytoscape, built on a complex database that integrates interactions among human phenotypes, proteins, tissues, and drugs. It utilizes the power of multi-partite network analysis and visualization to uncover genetic similarities among multiple traits to suggest alternative therapeutic approaches and to prioritize disease-associated genes. iCTNet enables a point and click environment to load views for user-selected phenotypes, and provides two methods for evaluation or prioritization of disease-causing genes. To maintain iCTNet, monthly updates of GWAS catalog are planned. Integration of further data sources including quantitative omics data, miRNA targets, and advanced analysis are among future plans.

## Availability and Requirements

• Project name: iCTNet, integrated Complex Traits Networks.

• Project home page: http://www.cs.queensu.ca/ictnet

• Operating system: Platform independent

• Programming language: Java, minimum requirement Java SE 1.5

• Cytoscape version: iCTNet requires Cytoscape version 2.6 or later, and has been tested on version 2.8

• Memory: minimum 2GB for large networks

• License: BSD-style open source license

• Any restrictions to use by non-academics: none other than those in the BSD license

## List of abbreviations

iCTNet: integrated complex trait networks; SNP: single nucleotide polymorphism; GWAS: genome-wide association study; HPRD: Human protein reference database; CD: Crohn's disease; MS: multiple sclerosis; Ps: Psoriasis; T1D: Type 1 diabetes; RA: Rheumatoid arthritis

## Authors' contributions

SEB conceived the idea for the plugin. PM and LW developed the concept and LW programmed the database and the plugin. PK assisted with development. SEB, PM, LW, and PK tested the application. SEB and PM wrote the manuscript. all authors read and approved the final manuscript
